# Ethyl 2-[3-(4-nitro­benzo­yl)thio­ureido]benzoate

**DOI:** 10.1107/S1600536810011116

**Published:** 2010-03-31

**Authors:** Sohail Saeed, Naghmana Rashid, Wing-Tak Wong

**Affiliations:** a Department of Chemistry, Research Complex, Allama Iqbal Open University, Islamabad, Pakistan; b Department of Applied Biology and Chemical Technology, The Hong Kong Polytechnic University, Hung Hom, Kowloon, Hong Kong SAR, People’s Republic of China

## Abstract

In the title compound, C_17_H_15_N_3_O_5_S, the nitro and thio­ureido groups are twisted by 7.2 (7) and 21.4 (2)°, respectively, from the nitro­benzene ring plane whereas the thio­ureido and the ethyl ester group make dihedral angles of 43.0 (1) and 18.0 (2)°, respectively, with the benzene rings to which they are attached. Intra­molecular N—H⋯O hydrogen-bonding inter­actions are observed. In the crystal, inter­molecular N—H⋯O hydrogen bonds connect the mol­ecules into chains running along the *a* axis.

## Related literature

For general background to the chemistry of thio­urea derivatives, see: Ugur *et al.* (2006 ▶). For related compounds with anti­tubercular properties, see: Huebner *et al.* (1953 ▶) and for other biological activities of thio­urea compounds, see: Glasser & Doughty (1964 ▶). For related structures, see: Saeed *et al.* (2008*a* ▶,*b* ▶). For the cytotoxicity of anti­cancer drugs to normal cells in cancer therapy, see: Saeed *et al.* (2010 ▶). For the herbicidal activity of thio­urea derivatives, see: Zheng *et al.* (2004 ▶).
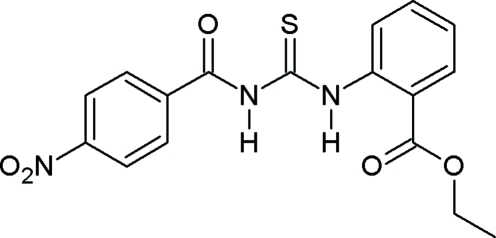


## Experimental

### 

#### Crystal data

C_17_H_15_N_3_O_5_S
*M* *_r_* = 373.38
Orthorhombic, 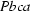

*a* = 9.0698 (13) Å
*b* = 15.778 (2) Å
*c* = 24.889 (4) Å
*V* = 3561.7 (9) Å^3^
*Z* = 8
Mo *K*α radiation
μ = 0.22 mm^−1^
*T* = 295 K
0.36 × 0.25 × 0.03 mm

#### Data collection

Bruker SMART 1000 CCD diffractometer
Absorption correction: multi-scan (*SADABS*; Sheldrick, 1996 ▶) *T*_min_ = 0.927, *T*_max_ = 0.994
23121 measured reflections
4362 independent reflections
2877 reflections with *I* > 2σ(*I*)
*R*_int_ = 0.032

#### Refinement

*R*[*F* ^2^ > 2σ(*F* ^2^)] = 0.059
*wR*(*F* ^2^) = 0.171
*S* = 1.07
4362 reflections
244 parameters
6 restraints
H atoms treated by a mixture of independent and constrained refinement
Δρ_max_ = 0.63 e Å^−3^
Δρ_min_ = −0.32 e Å^−3^

### 

Data collection: *SMART* (Bruker, 1998 ▶); cell refinement: *SAINT* (Bruker, 2006 ▶); data reduction: *SAINT* and *CrystalStructure* (Rigaku/MSC and Rigaku, 2006 ▶); program(s) used to solve structure: *SHELXS97* (Sheldrick, 2008 ▶); program(s) used to refine structure: *SHELXL97* (Sheldrick, 2008 ▶); molecular graphics: *ORTEPII* (Johnson, 1976 ▶); software used to prepare material for publication: *SHELXL97*.

## Supplementary Material

Crystal structure: contains datablocks global, I. DOI: 10.1107/S1600536810011116/si2248sup1.cif

Structure factors: contains datablocks I. DOI: 10.1107/S1600536810011116/si2248Isup2.hkl

Additional supplementary materials:  crystallographic information; 3D view; checkCIF report

## Figures and Tables

**Table 1:** Hydrogen-bond geometry (Å, °)

*D*—H⋯*A*	*D*—H	H⋯*A*	*D*⋯*A*	*D*—H⋯*A*
N2—H2*N*⋯O3^i^	0.80 (4)	2.12 (4)	2.903 (3)	165 (3)
N3—H3*N*⋯O3	0.91 (3)	1.91 (3)	2.664 (3)	139 (3)
N3—H3*N*⋯O4	0.91 (3)	2.15 (3)	2.721 (3)	120 (2)

Symmetry code: (i) 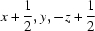.

**Fig. 1.**
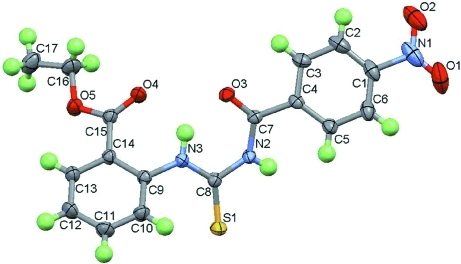
The ORTEP plot of the compound was shown at 30% probability thermal ellipsoids with the atom numbering scheme.

**Fig. 2.**
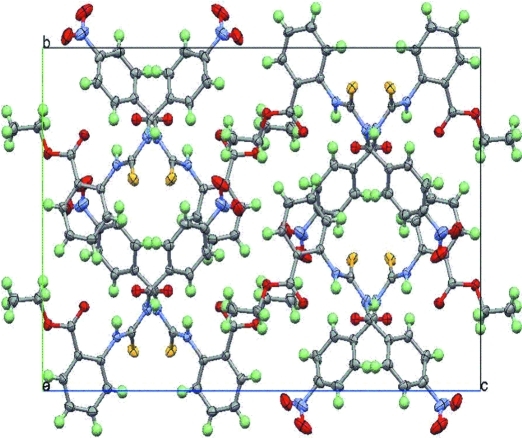
The unit cell packing diagram of the compound was projected down the a-axis and shown at 30% probability thermal ellipsoids.

## supplementary crystallographic information

### Comment

Industrial production and the use of transition elements can cause environmental pollution. However, some of these metals are present in trace amounts as essential elements for biological systems and also play an important role in bioinorganic chemistry. In order to understand the role of these metal ions in biological systems, structural studies of the biological compounds and their metal complexes are extremely important. Compounds containing carbonyl and thiocarbonyl groups occupy an important position among organic reagents as potential donor ligands for transition metal ions (Ugur *et al.*, 2006). Thioureas are also known to exhibit a wide range of biological activities including antiviral, antibacterial, anticancer (Saeed *et al.*, 2010), antifungal, antitubercular, antithyroidal, herbicidal and insecticidal activities (Huebner *et al.*, 1953) and as agrochemicals (Saeed *et al.*, 2008a). An example is furnished by 1- benzoyl-3-(4,5-disubstituted-pyrimidine-2-yl)- thioureas, which have excellent herbicidal activity (Zheng *et al.*, 2004). Thioureas are also well known chelating agents for transition metals and the complexes also show varied biological activities (Glasser & Doughty, 1964). Thioureas and substituted thioureas are also known as epoxy resin curing agents (Saeed *et al.*, 2008b). We became interested in the synthesis of N-aroyl, *N*'-arylthioureas as intermediates towards some new novel heterocycles and for the systematic study of their bioactive complexes and their function as epoxy resin curing agents. Here we present the structure of the title compound (I). The molecule is not planar. The nitro group is slightly twisted (7.2 (7)°) from the benzene ring plane of C1—C6. The thioureido group is 21.4 (1)° from the benzene ring plane of C1—C6 and 43.0 (1)° from the benzene ring plane of C9—C14. The ethyl ester group is twisted 18.0 (2)° from the benzene ring plane of C9—C14. Both intra- and inter-molecular N—H···O H-bond interactions are observed in the crystal lattice. The intermolecular N2—H2N···O3 H-bonding interactions, connect the molecules into 1-D chains running along the a-axis. There seems to be no significant π···π nor C—H···π interaction in the crystal lattice.

There is no residual solvent accessible void volume in the unit cell.

### Experimental

A solution of 4-nitrobenzoyl chloride (0.01 mol) in dry acetone (80 ml) was added dropwise to a suspension of ammonium thiocyanate (0.01 mol) in acetone (50 ml) and the reaction mixture was refluxed for 45 minutes. After cooling to room temperature, a solution of ethyl 2-aminobenzoate (0.01 mol) in acetone (25 ml) was added and the resulting mixture refluxed for 2 h. The reaction mixture was poured into five times its volume of cold water, upon which the thiourea precipitated. The product was recrystallized from ethyl acetate as intensely yellow crystals.

### Refinement

All of the C-bound H atoms are observable from difference Fourier map but are all placed at geometrical positions with C—H = 0.93, 0.96 and 0.97Å for phenyl methyl and methylene H-atoms. All C-bound H-atoms are refined using riding model with *U*_iso_(H) = 1.2*U*_eq_(Carrier).

The N-bound H atoms are located from a difference Fourier map and refined isotropically. Six restraints are related to the refinement of O2 using isotropic restraints of standard deviation of 0.001 in the anisotropic atom displacement components.

Highest peak is 0.63 at (0.3420, 0.1232, 0.3759) [0.84Å from O2] Deepest hole is -0.32 at (0.3460, 0.0919, 0.4004) [0.36Å from O2]

### Crystal data

**Table:**

C_17_H_15_N_3_O_5_S	*D*_x_ = 1.393 Mg m^−^^3^
*M**_r_* = 373.38	Melting point: 438 K
Orthorhombic, *P**b**c**a*	Mo *K*α radiation, λ = 0.71073 Å
Hall symbol: -P 2ac 2ab	Cell parameters from 23121 reflections
*a* = 9.0698 (13) Å	θ = 1.6–28.3°
*b* = 15.778 (2) Å	µ = 0.22 mm^−^^1^
*c* = 24.889 (4) Å	*T* = 295 K
*V* = 3561.7 (9) Å^3^	Plate, yellow
*Z* = 8	0.36 × 0.25 × 0.03 mm
*F*(000) = 1552	

### Data collection

**Table:**

Bruker SMART 1000 CCD diffractometer	4362 independent reflections
Radiation source: fine-focus sealed tube	2877 reflections with *I* > 2σ(*I*)
graphite	*R*_int_ = 0.032
ω scan	θ_max_ = 28.3°, θ_min_ = 1.6°
Absorption correction: multi-scan (*SADABS*; Sheldrick, 1996)	*h* = −8→11
*T*_min_ = 0.927, *T*_max_ = 0.994	*k* = −21→21
23121 measured reflections	*l* = −33→32

### Refinement

**Table:**

Refinement on *F*^2^	Primary atom site location: structure-invariant direct methods
Least-squares matrix: full	Secondary atom site location: difference Fourier map
*R*[*F*^2^ > 2σ(*F*^2^)] = 0.059	Hydrogen site location: inferred from neighbouring sites
*wR*(*F*^2^) = 0.171	H atoms treated by a mixture of independent and constrained refinement
*S* = 1.07	*w* = 1/[σ^2^(*F*_o_^2^) + (0.0709*P*)^2^ + 2.2491*P*] where *P* = (*F*_o_^2^ + 2*F*_c_^2^)/3
4362 reflections	(Δ/σ)_max_ < 0.001
244 parameters	Δρ_max_ = 0.63 e Å^−^^3^
6 restraints	Δρ_min_ = −0.32 e Å^−^^3^

### Special details

**Table:**

Geometry. All esds (except the esd in the dihedral angle between two l.s. planes) are estimated using the full covariance matrix. The cell esds are taken into account individually in the estimation of esds in distances, angles and torsion angles; correlations between esds in cell parameters are only used when they are defined by crystal symmetry. An approximate (isotropic) treatment of cell esds is used for estimating esds involving l.s. planes.
Refinement. Refinement of *F*^2^ against ALL reflections. The weighted *R*-factor wR and goodness of fit *S* are based on *F*^2^, conventional *R*-factors *R* are based on *F*, with *F* set to zero for negative *F*^2^. The threshold expression of *F*^2^ > σ(*F*^2^) is used only for calculating *R*-factors(gt) etc. and is not relevant to the choice of reflections for refinement. *R*-factors based on *F*^2^ are statistically about twice as large as those based on *F*, and *R*- factors based on ALL data will be even larger.

### Fractional atomic coordinates and isotropic or equivalent isotropic displacement parameters (Å^2^)

**Table:**

	*x*	*y*	*z*	*U*_iso_*/*U*_eq_	
S1	0.51557 (9)	0.11637 (5)	0.20685 (4)	0.0587 (3)	
O1	0.3603 (6)	0.5330 (3)	0.44429 (14)	0.1331 (16)	
O2	0.1862 (4)	0.6034 (2)	0.40527 (18)	0.1245 (13)	
O3	0.1165 (2)	0.29106 (13)	0.21392 (8)	0.0476 (5)	
O4	0.0879 (3)	0.24551 (13)	0.09489 (9)	0.0641 (6)	
O5	0.0925 (3)	0.19226 (14)	0.01211 (9)	0.0662 (7)	
N1	0.2655 (5)	0.5415 (2)	0.41024 (19)	0.0936 (13)	
N2	0.3396 (2)	0.23826 (15)	0.23814 (10)	0.0428 (5)	
N3	0.2463 (3)	0.15722 (15)	0.16898 (9)	0.0414 (5)	
C1	0.2484 (4)	0.4741 (2)	0.36882 (15)	0.0648 (9)	
C2	0.1512 (4)	0.4866 (2)	0.32689 (17)	0.0690 (10)	
H2	0.0937	0.5353	0.3250	0.083*	
C3	0.1416 (4)	0.4242 (2)	0.28770 (13)	0.0552 (8)	
H3	0.0758	0.4306	0.2593	0.066*	
C4	0.2296 (3)	0.35235 (17)	0.29062 (11)	0.0423 (6)	
C5	0.3214 (3)	0.3409 (2)	0.33479 (12)	0.0516 (7)	
H5	0.3764	0.2914	0.3379	0.062*	
C6	0.3312 (4)	0.4026 (2)	0.37399 (13)	0.0630 (9)	
H6	0.3932	0.3955	0.4034	0.076*	
C7	0.2219 (3)	0.29142 (16)	0.24481 (10)	0.0388 (6)	
C8	0.3576 (3)	0.17016 (17)	0.20286 (10)	0.0411 (6)	
C9	0.2299 (3)	0.08868 (17)	0.13261 (11)	0.0423 (6)	
C10	0.2679 (4)	0.00680 (19)	0.14754 (13)	0.0575 (8)	
H10	0.3060	−0.0030	0.1817	0.069*	
C11	0.2504 (4)	−0.0600 (2)	0.11286 (15)	0.0661 (9)	
H11	0.2767	−0.1144	0.1236	0.079*	
C12	0.1938 (4)	−0.0466 (2)	0.06204 (15)	0.0659 (9)	
H12	0.1837	−0.0916	0.0382	0.079*	
C13	0.1526 (4)	0.03375 (19)	0.04696 (13)	0.0552 (8)	
H13	0.1138	0.0424	0.0128	0.066*	
C14	0.1677 (3)	0.10259 (17)	0.08161 (11)	0.0435 (6)	
C15	0.1132 (3)	0.18718 (18)	0.06483 (12)	0.0472 (7)	
C16	0.0290 (5)	0.2705 (2)	−0.00865 (15)	0.0782 (11)	
H16A	0.0907	0.3184	0.0010	0.094*	
H16B	−0.0684	0.2794	0.0064	0.094*	
C17	0.0198 (5)	0.2627 (3)	−0.06722 (17)	0.0872 (13)	
H17A	−0.0220	0.3135	−0.0820	0.105*	
H17B	−0.0414	0.2152	−0.0763	0.105*	
H17C	0.1168	0.2543	−0.0817	0.105*	
H2N	0.409 (4)	0.2503 (19)	0.2566 (13)	0.047 (9)*	
H3N	0.177 (3)	0.200 (2)	0.1694 (12)	0.050 (8)*	

### Atomic displacement parameters (Å^2^)

**Table:**

	*U*^11^	*U*^22^	*U*^33^	*U*^12^	*U*^13^	*U*^23^
S1	0.0427 (4)	0.0648 (5)	0.0686 (5)	0.0143 (3)	−0.0073 (4)	−0.0180 (4)
O1	0.208 (5)	0.116 (3)	0.075 (2)	−0.037 (3)	−0.004 (3)	−0.037 (2)
O2	0.099 (2)	0.104 (2)	0.171 (3)	−0.0053 (18)	0.0240 (19)	−0.073 (2)
O3	0.0353 (10)	0.0564 (12)	0.0511 (11)	0.0026 (8)	−0.0027 (8)	0.0000 (9)
O4	0.0893 (17)	0.0445 (12)	0.0584 (13)	0.0126 (11)	−0.0185 (12)	−0.0062 (10)
O5	0.0994 (19)	0.0516 (13)	0.0476 (12)	0.0076 (12)	−0.0137 (12)	0.0037 (10)
N1	0.094 (3)	0.073 (2)	0.114 (3)	−0.026 (2)	0.042 (2)	−0.039 (2)
N2	0.0302 (11)	0.0540 (14)	0.0442 (13)	0.0015 (10)	−0.0021 (10)	−0.0106 (10)
N3	0.0400 (12)	0.0416 (12)	0.0425 (12)	0.0015 (10)	−0.0035 (10)	−0.0026 (10)
C1	0.065 (2)	0.062 (2)	0.067 (2)	−0.0163 (17)	0.0258 (18)	−0.0232 (17)
C2	0.066 (2)	0.0466 (18)	0.094 (3)	0.0033 (15)	0.027 (2)	−0.0093 (18)
C3	0.0485 (17)	0.0510 (17)	0.0661 (19)	0.0060 (13)	0.0097 (15)	0.0010 (15)
C4	0.0356 (13)	0.0448 (14)	0.0466 (14)	−0.0019 (11)	0.0115 (11)	−0.0026 (12)
C5	0.0468 (16)	0.0601 (18)	0.0479 (16)	0.0042 (13)	0.0045 (13)	−0.0090 (14)
C6	0.058 (2)	0.083 (2)	0.0482 (17)	−0.0072 (18)	0.0094 (15)	−0.0178 (16)
C7	0.0310 (12)	0.0442 (14)	0.0410 (13)	−0.0020 (10)	0.0057 (11)	0.0024 (11)
C8	0.0379 (13)	0.0476 (15)	0.0378 (13)	−0.0009 (11)	0.0031 (11)	−0.0008 (11)
C9	0.0416 (14)	0.0404 (14)	0.0448 (14)	−0.0014 (11)	0.0001 (12)	−0.0015 (11)
C10	0.068 (2)	0.0458 (16)	0.0589 (19)	0.0016 (15)	−0.0138 (16)	0.0046 (14)
C11	0.079 (2)	0.0407 (17)	0.079 (2)	0.0046 (16)	−0.0134 (19)	0.0007 (15)
C12	0.086 (3)	0.0419 (17)	0.070 (2)	0.0032 (16)	−0.0107 (19)	−0.0121 (15)
C13	0.067 (2)	0.0484 (17)	0.0496 (16)	0.0006 (14)	−0.0096 (15)	−0.0070 (13)
C14	0.0460 (15)	0.0416 (14)	0.0429 (14)	−0.0026 (11)	−0.0026 (12)	−0.0010 (11)
C15	0.0489 (16)	0.0447 (16)	0.0481 (16)	−0.0025 (12)	−0.0088 (13)	0.0004 (12)
C16	0.107 (3)	0.059 (2)	0.069 (2)	0.012 (2)	−0.021 (2)	0.0121 (18)
C17	0.103 (3)	0.083 (3)	0.076 (3)	−0.005 (2)	−0.026 (2)	0.021 (2)

### Geometric parameters (Å, °)

**Table:**

S1—C8	1.668 (3)	C4—C7	1.493 (4)
O1—N1	1.215 (6)	C5—C6	1.381 (4)
O2—N1	1.219 (5)	C5—H5	0.9300
O3—C7	1.227 (3)	C6—H6	0.9300
O4—C15	1.208 (3)	C9—C10	1.388 (4)
O5—C15	1.328 (3)	C9—C14	1.406 (4)
O5—C16	1.457 (4)	C10—C11	1.371 (4)
N1—C1	1.489 (5)	C10—H10	0.9300
N2—C7	1.368 (3)	C11—C12	1.381 (5)
N2—C8	1.397 (3)	C11—H11	0.9300
N2—H2N	0.80 (3)	C12—C13	1.373 (5)
N3—C8	1.331 (3)	C12—H12	0.9300
N3—C9	1.418 (3)	C13—C14	1.394 (4)
N3—H3N	0.91 (3)	C13—H13	0.9300
C1—C6	1.361 (5)	C14—C15	1.483 (4)
C1—C2	1.380 (6)	C16—C17	1.465 (6)
C2—C3	1.388 (5)	C16—H16A	0.9700
C2—H2	0.9300	C16—H16B	0.9700
C3—C4	1.388 (4)	C17—H17A	0.9600
C3—H3	0.9300	C17—H17B	0.9600
C4—C5	1.391 (4)	C17—H17C	0.9600
			
C15—O5—C16	117.2 (3)	C10—C9—C14	119.1 (3)
O1—N1—O2	125.2 (4)	C10—C9—N3	120.9 (3)
O1—N1—C1	118.6 (4)	C14—C9—N3	119.9 (2)
O2—N1—C1	116.1 (5)	C11—C10—C9	121.2 (3)
C7—N2—C8	129.7 (2)	C11—C10—H10	119.4
C7—N2—H2N	113 (2)	C9—C10—H10	119.4
C8—N2—H2N	117 (2)	C10—C11—C12	120.1 (3)
C8—N3—C9	126.9 (2)	C10—C11—H11	119.9
C8—N3—H3N	113.5 (19)	C12—C11—H11	119.9
C9—N3—H3N	119.5 (19)	C13—C12—C11	119.5 (3)
C6—C1—C2	122.8 (3)	C13—C12—H12	120.2
C6—C1—N1	117.9 (4)	C11—C12—H12	120.2
C2—C1—N1	119.2 (4)	C12—C13—C14	121.6 (3)
C1—C2—C3	118.0 (3)	C12—C13—H13	119.2
C1—C2—H2	121.0	C14—C13—H13	119.2
C3—C2—H2	121.0	C13—C14—C9	118.4 (3)
C2—C3—C4	120.4 (3)	C13—C14—C15	119.6 (3)
C2—C3—H3	119.8	C9—C14—C15	121.9 (2)
C4—C3—H3	119.8	O4—C15—O5	122.6 (3)
C3—C4—C5	119.4 (3)	O4—C15—C14	125.1 (3)
C3—C4—C7	117.3 (3)	O5—C15—C14	112.3 (2)
C5—C4—C7	123.2 (2)	O5—C16—C17	107.7 (3)
C6—C5—C4	120.4 (3)	O5—C16—H16A	110.2
C6—C5—H5	119.8	C17—C16—H16A	110.2
C4—C5—H5	119.8	O5—C16—H16B	110.2
C1—C6—C5	118.8 (3)	C17—C16—H16B	110.2
C1—C6—H6	120.6	H16A—C16—H16B	108.5
C5—C6—H6	120.6	C16—C17—H17A	109.5
O3—C7—N2	122.0 (2)	C16—C17—H17B	109.5
O3—C7—C4	121.2 (2)	H17A—C17—H17B	109.5
N2—C7—C4	116.8 (2)	C16—C17—H17C	109.5
N3—C8—N2	115.3 (2)	H17A—C17—H17C	109.5
N3—C8—S1	127.7 (2)	H17B—C17—H17C	109.5
N2—C8—S1	117.0 (2)		

### Hydrogen-bond geometry (Å, °)

**Table:**

*D*—H···*A*	*D*—H	H···*A*	*D*···*A*	*D*—H···*A*
N2—H2N···O3^i^	0.80 (4)	2.12 (4)	2.903 (3)	165 (3)
N3—H3N···O3	0.91 (3)	1.91 (3)	2.664 (3)	139 (3)
N3—H3N···O4	0.91 (3)	2.15 (3)	2.721 (3)	120 (2)

Symmetry codes: (i) *x*+1/2, *y*, −*z*+1/2.

## References

[bb1] Bruker (1998). *SMART* Bruker AXS Inc., Madison, Wisconsin, USA.

[bb2] Bruker (2006). *SAINT* Bruker AXS Inc., Madison, Wisconsin, USA.

[bb3] Glasser, A. C. & Doughty, R. M. (1964). *J. Pharm. Soc.* **53**, 40–42. 10.1002/jps.2600530107 14106372

[bb4] Huebner, C. F., Marsh, J. L., Mizzoni, R. H., Mull, R. P., Schroeder, D. C., Troxell, H. A. & Scholz, C. R. (1953). *J. Am. Chem. Soc.* **75**, 2274–2275.

[bb5] Johnson, C. K. (1976). *ORTEPII* Report ORNL-5138. Oak Ridge National Laboratory, Tennessee, USA.

[bb6] Rigaku/MSC and Rigaku (2006). *CrystalStructure* Rigaku/MSC, The Woodlands, Texas, USA, and Rigaku Corporation, Tokyo, Japan.

[bb7] Saeed, S., Bhatti, M. H., Tahir, M. K. & Jones, P. G. (2008*a*). *Acta Cryst.* E**64**, o1369. 10.1107/S1600536808017868 PMC2961839 21202987

[bb8] Saeed, S., Bhatti, M. H., Yunus, U. & Jones, P. G. (2008*b*). *Acta Cryst.* E**64**, o1485. 10.1107/S1600536808017856 PMC2962115 21203197

[bb9] Saeed, S., Rashid, N., Jones, P. G., Ali, M. & Hussain, R. (2010). *Eur. J. Med. Chem.* **45**, 1323–1331. 10.1016/j.ejmech.2009.12.016 20056520

[bb10] Sheldrick, G. M. (1996). *SADABS* University of Göttingen, Germany.

[bb11] Sheldrick, G. M. (2008). *Acta Cryst* A**64**, 112–122. 10.1107/S0108767307043930 18156677

[bb12] Ugur, D., Arslan, H. & Külcü, N. (2006). *Russ. J. Coord. Chem.* **32**, 669–675.

[bb13] Zheng, W., Yates, S. R., Papiernik, S. K. & Guo, M. (2004). *Environ. Sci. Technol.* **38**, 6855–6860. 10.1021/es049384+ 15669349

